# Supplementary material for: Omecamtiv mecarbil and Mavacamten target the same myosin pocket despite opposite effects in heart contraction
Source: Nat Commun. 2024 Jun 7;15:4885. doi: 10.1038/s41467-024-47587-9 (PMC11161628; doi:10.1038/s41467-024-47587-9)
Supplement: Supplementary file 1 — Supplementary information [file 41467_2024_47587_MOESM1_ESM.pdf]

Supplementary information

Omecamtiv mecarbil and Mavacamten target the same myosin pocket despite opposite effects in heart contraction

Daniel Auguin*, Julien Robert-Paganin*, Stéphane Réty, Carlos Kikuti, Amandine David, Gabriele Theumer, Arndt W. Schmidt, Hans-Joachim Knölker, Anne Houdusse.

SUMMARY

Supplementary Figures 1-11 pages 2-12

Supplementary Tables 1-3 pages 13-15

Supplementary References pages 16

Supplementary Figures:

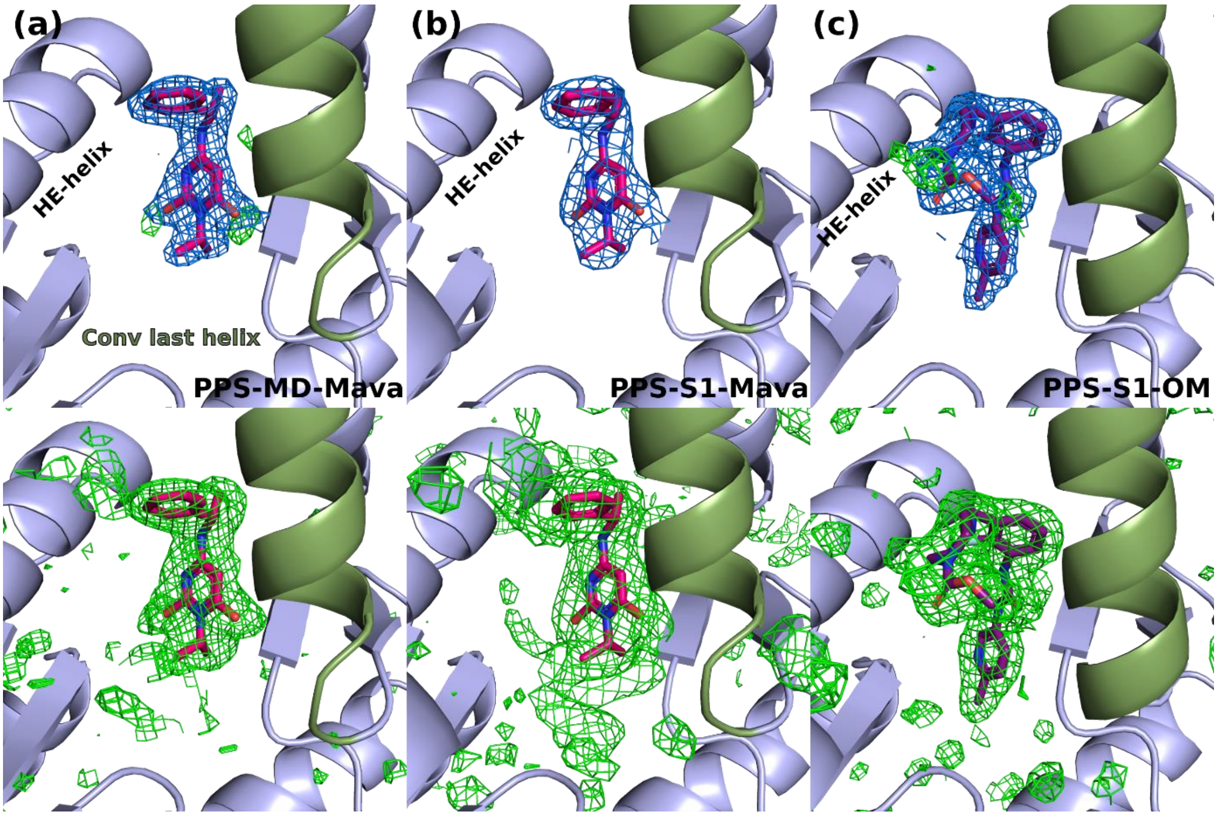

<u>Supplementary Figure 1 – The drugs are defined without ambiguity in the electron density maps</u>. The three panels present the electron density maps corresponding to the drugs bound in the three β-cardiac myosin structures: **(a)** PPS-MD-Mava; **(b)** PPS-S1-Mava and **(c)** PPS-S1-OM. Upper panel: the 2Fo-Fc map contoured at 1 σ is represented in blue; the positive peaks of difference map Fo-Fc, contoured at 3 σ, are represented in green. The last helix of the Converter is colored in smudge green and labelled as "Conv last helix". Lower panel: Polder omit map[1] contoured at 3.0 σ (green) calculated with the drug as omit selection. In the three cases, the drug is present without ambiguity.

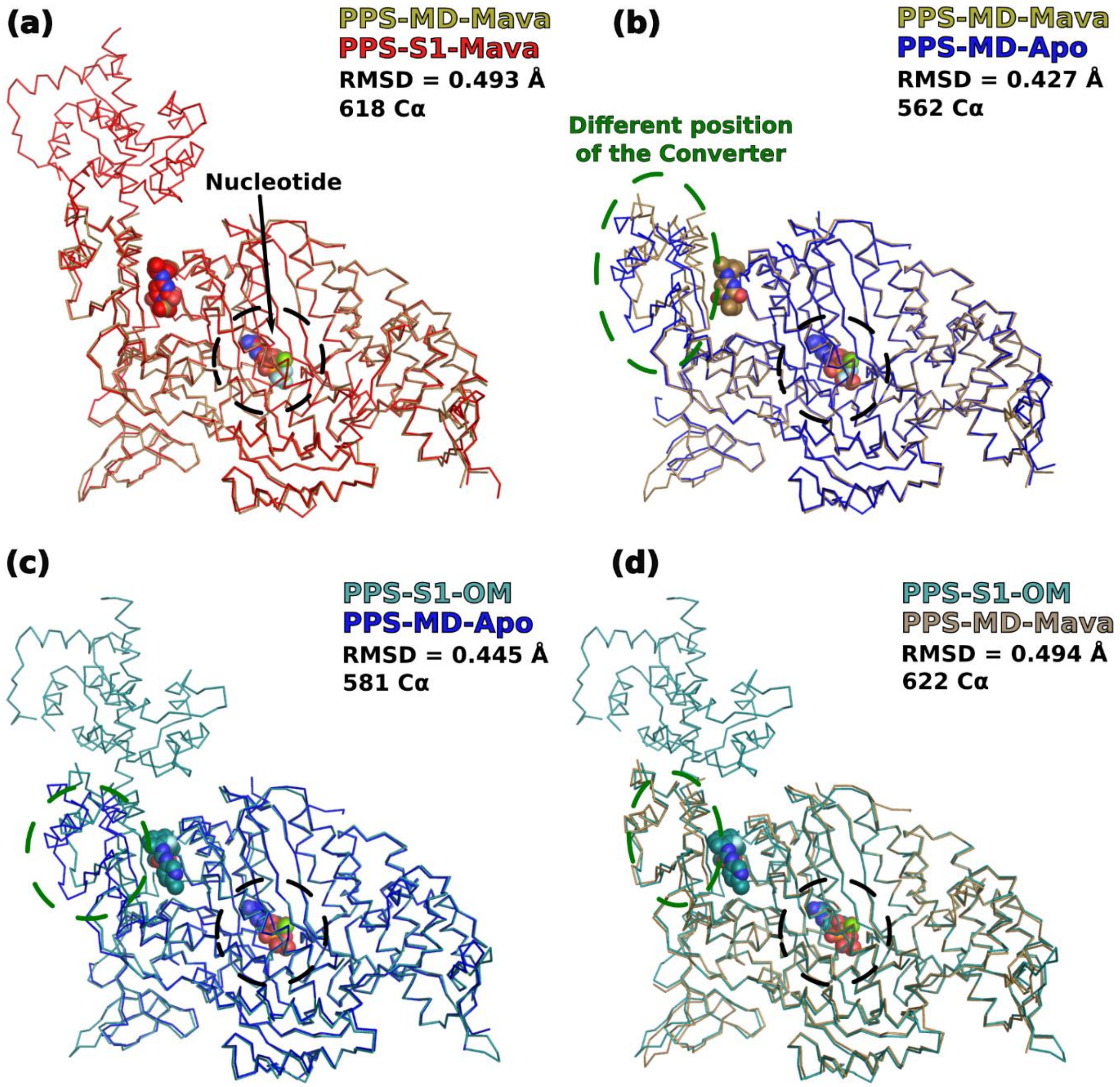

Supplementary Figure 2 – Comparison of the different β-cardiac myosin PPS structures from this work. **(a)** Superimposition of the PPS-MD-Mava and PPS-S1-Mava chain A; **(b)** PPS-MD-Mava and PPS-MD-Apo; **(c)** PPS-S1-OM chain A and PPS-MD-Apo; **(d)** PPS-S1-OM chain A and PPS-MD-Mava. The RMSD on Cα is indicated as well as the colors used for each of these structures. The alignment was performed using a selection of the Cα in the motor domain (residues (1-780)). The drugs and the nucleotide are represented in spheres. The nucleotide position is contoured in black with dashed lines. When the position of the Converter differs significantly between the structures compared, the Converter position is contoured with green dashed lines.

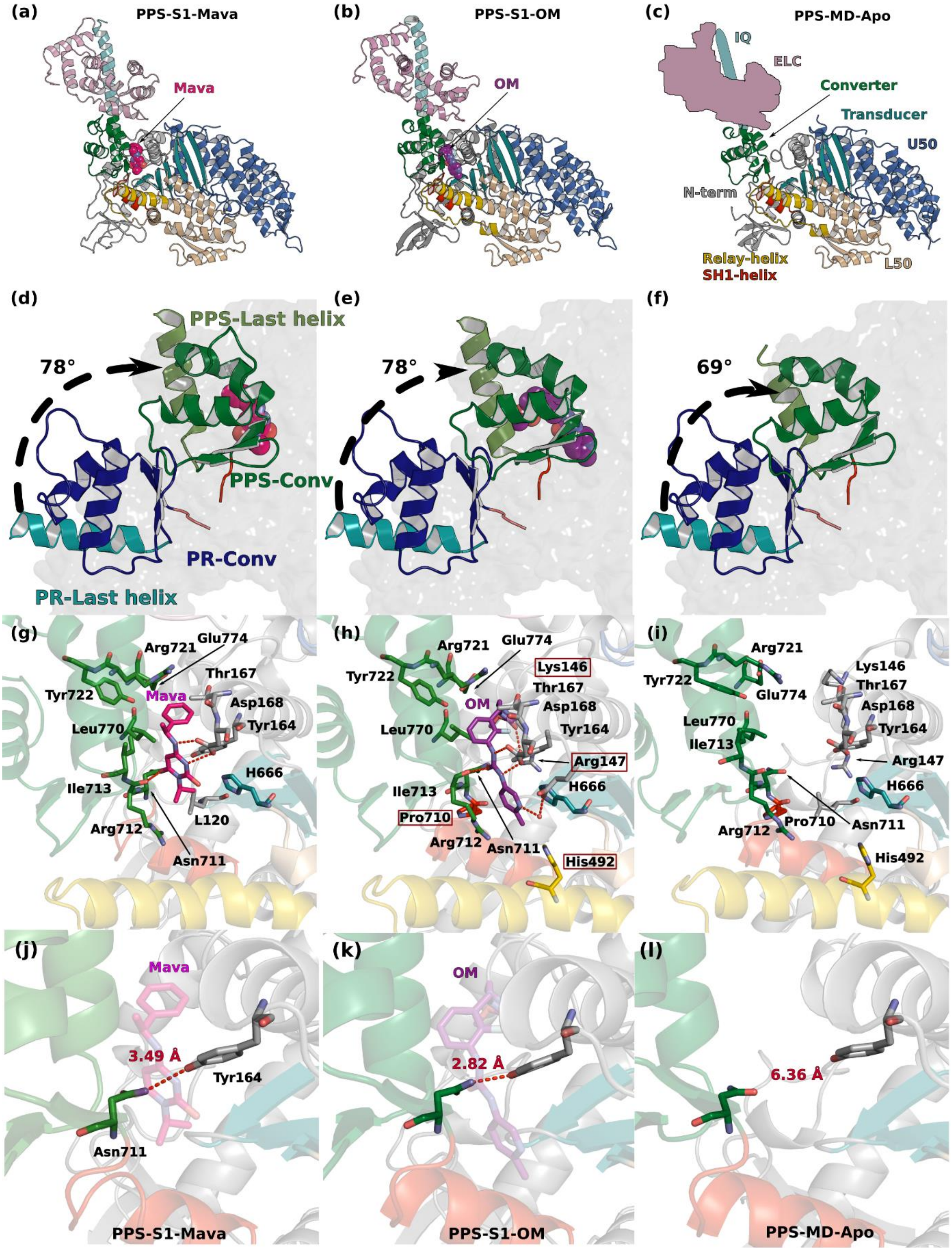

__Supplementary Figure 3 – OM and Mava have distinct effects on the priming of the Lever arm__. **(a), (b), (c)** Overall structures of PPS-S1-Mava, PPS-S1-OM and PPS-MD-Apo. **(d)**, **(e)** and **(f)**, Comparison of the priming of the Lever arm, while the structures are superimposed on the first 707 residues of the motor domain**.** The angle is measured between the PPS and the post-rigor structure (PDB code 6FSA[2]) using the position of the last helix of the Converter (769-780). **(g)**, **(h)**, **(i)** Zooms on the OM/Mava binding pocket. **(j)**, **(k)**, **(l)** Effects of drug binding on pocket size.

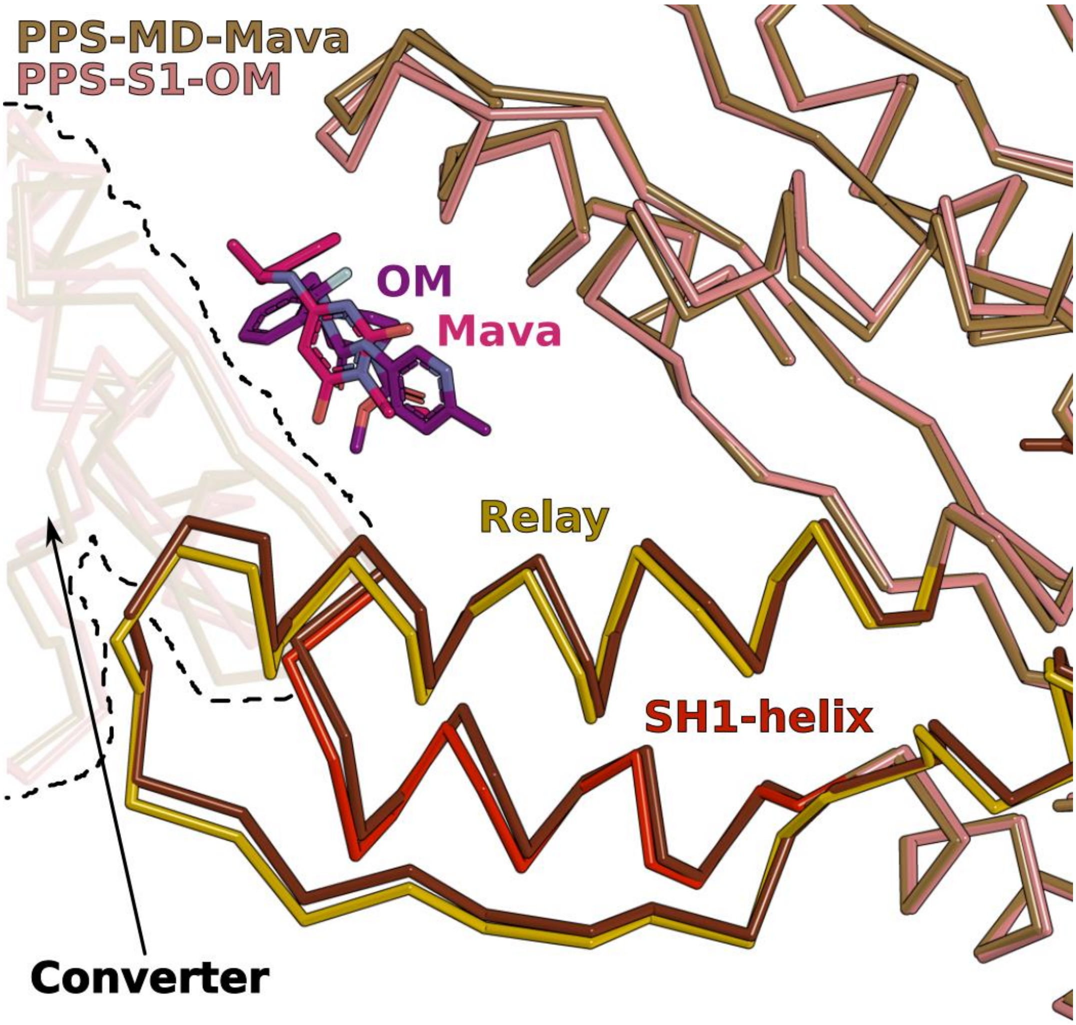

Supplementary Figure 4 – Comparison of the pocket size when OM and Mava are bound. The PPS-S1-Mava and PPS-S1-OM structures are superimposed on the N-term subdomains. The position of the Relay and SH1-helices differ in the two PPS structures when OM or Mava are bound. Note also in the back how these differences correspond to different positions of the Converter in the two structures (contoured with black dashed lines).

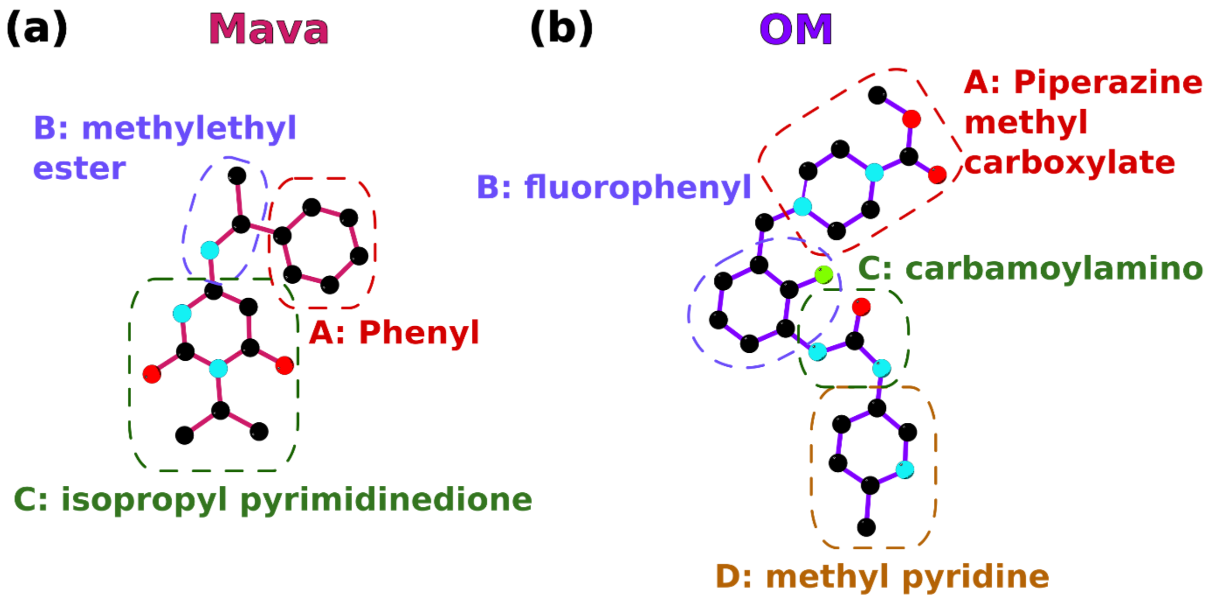

Supplementary Figure 5 – The different chemical groups of Mava and OM. (a) Schematic view of the chemical groups of Mavacamten: B: phenyl, B: methylethyl ester; C: isopropyl pyrimidinedione. **(b)** Schematic view of the chemical groups of Omecamtiv mecarbil: A: piperazine methyl carboxylate; B: fluorophenyl; C: carbamoylamino; D: methyl pyridine.

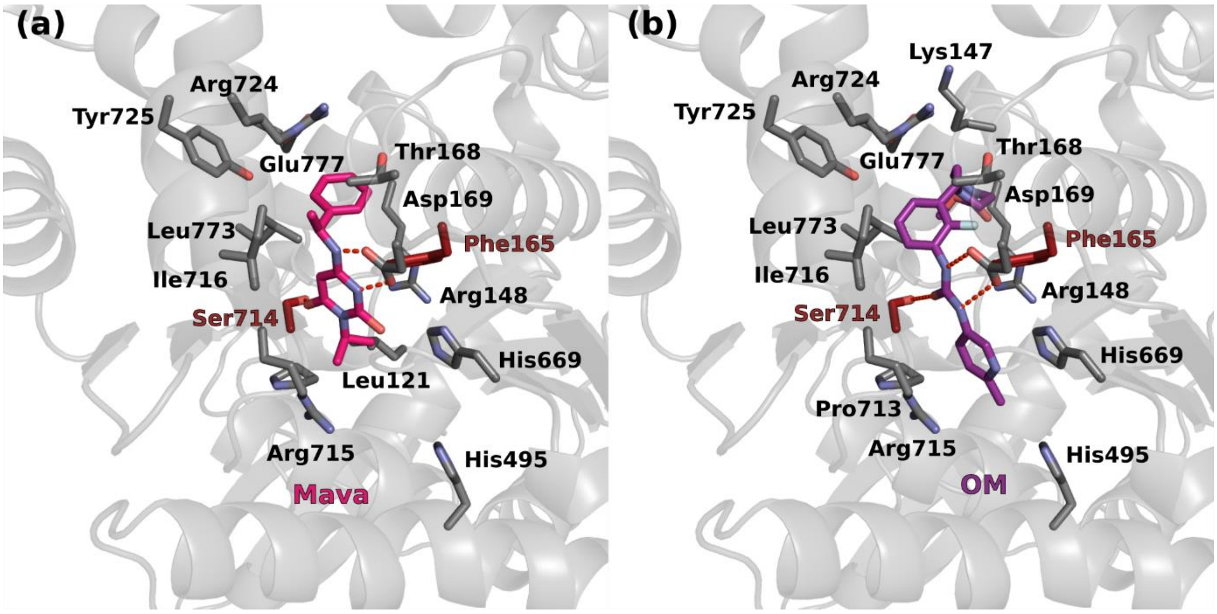

Supplementary Figure 6 – Homology model of Oryctolagus cuniculus skeletal myosin 2 (SkMyo2_Oc) in PPS bound to (a) Mava or (b) OM. The PPS-S1-OM structure was used as a template for the modelling. Different residues are colored in red.

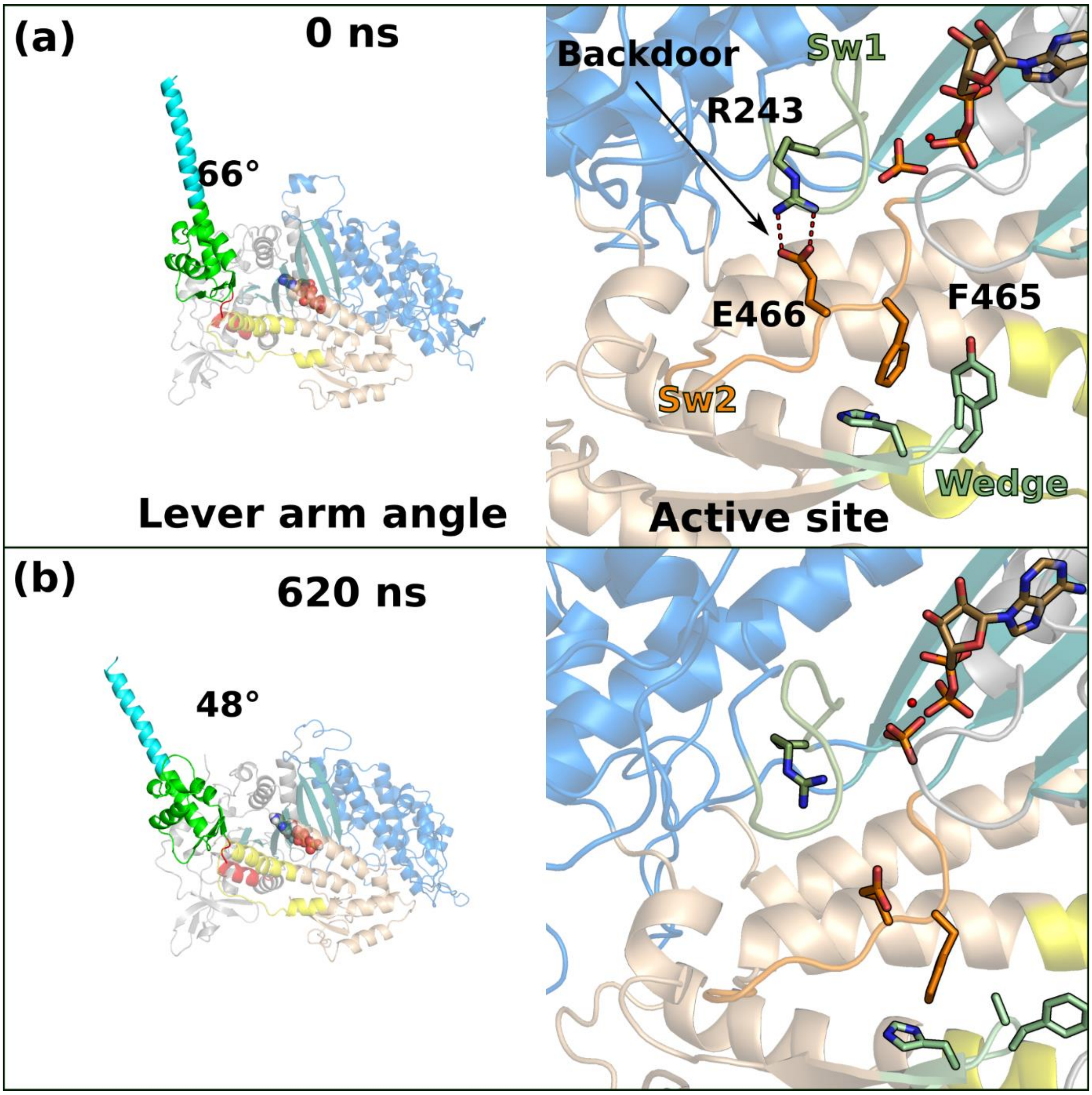

<u>Supplementary Figure 7 – All atom molecular dynamics of the S1 fragment in the apo form.</u> The consequences on the Lever arm angle (left) and on the active site (right) are represented. The subdomains and the connectors of the myosin are colored distinctly: Upper 50 kDa (U50) in dark blue, Lower 50 kDa (L50) in wheat, the Converter in green, the IQ domain in cyan, switch-2 (Sw2) in orange, switch-1 (Sw1) in smudge green, Wedge in pale green.

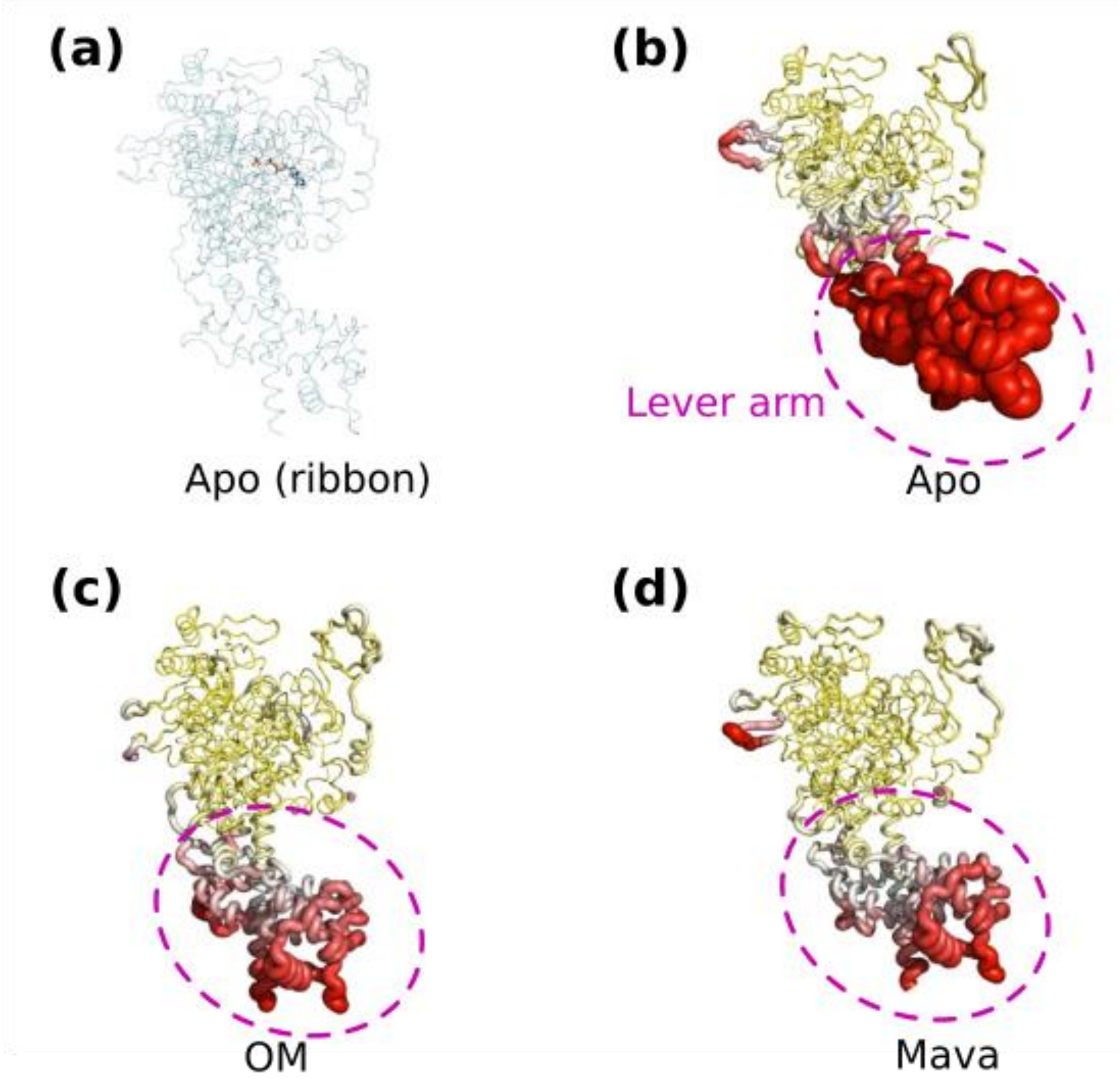

Supplementary figure 8 – Patterns of mobility for the different conditions starting from a similar conformation (PPS). (a) Apo S1-PPS + ADP.Pi hybrid model as visual reference. **(b)** "Putty representation" of the 730 ns trajectory of the Apo form. **(c)** "Putty representation" of the 1050ns trajectory of the OM form. **(d)** "Putty representation" of the 1050ns trajectory of the Mava form. All four subsets are depicted in the same referential. All "Putty representations' (using the 'psico module' PyMOL) are colored in yellow–white–red gradient with rmsd ranging from 0 to 9 Å. (Note that the maximum deviation for the Apo, OM and Mava MD simulations is 23.2 Å, 13.3 Å and 14.3 Å, respectively).

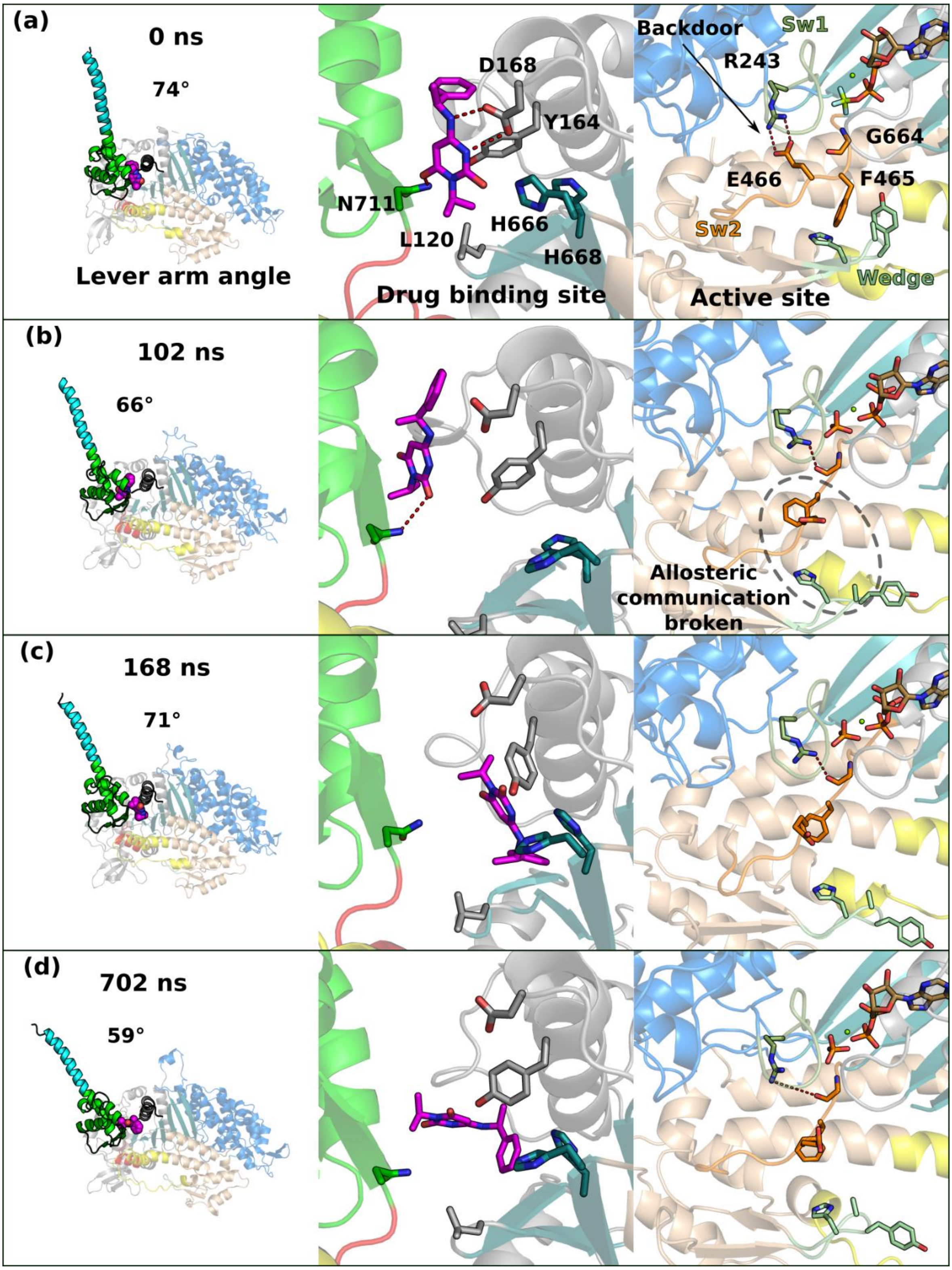

<u>Supplementary Figure 9 – All atom molecular dynamics of the S1 fragment bound to Mava.</u> (a), **(b), (c)** and **(d)** Representative times at which Mava occupies distinct positions in the pocket. The side chain of key residues are represented as sticks. The consequences on the Lever arm angle (left), on the pocket (center) and on the active site (right) are represented. The subdomains and the connectors of the myosin are colored distinctly: Upper 50 kDa (U50) in dark blue, Lower 50 kDa (L50) in wheat, the Converter in green, the IQ domain in cyan, switch-2 (Sw2) in orange, switch-1 (Sw1) in smudge green, Wedge in pale green.

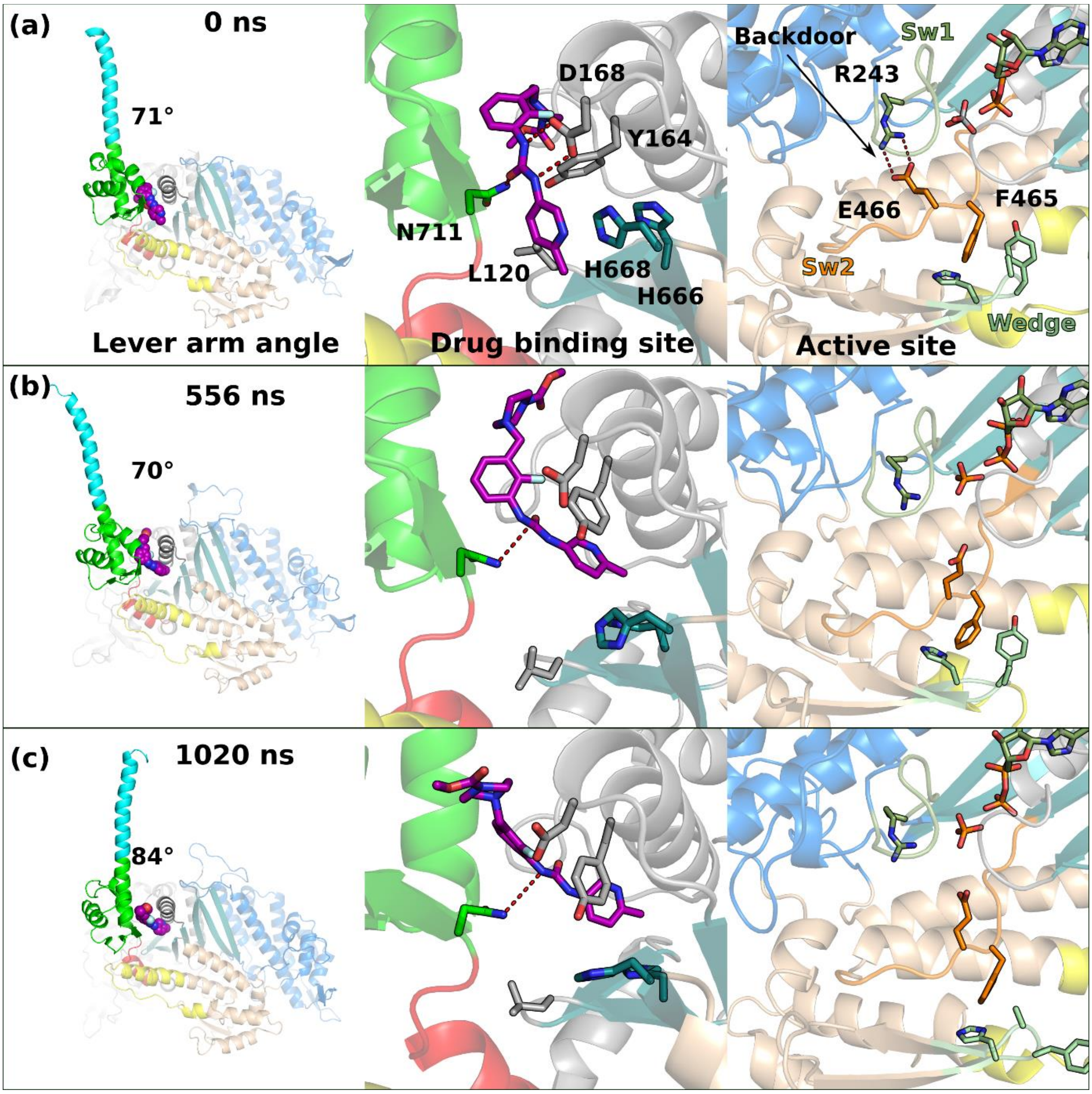

Supplementary Figure 10 – All atom molecular dynamics of the S1 fragment bound to OM. **(a)**, **(b)** and **(c)** Representative times at which OM occupies distinct positions in the pocket. The side chain of key residues are represented as sticks. The consequences on the Lever arm angle (left), on the pocket (center) and on the active site (right) are represented. The subdomains and the connectors of the myosin are colored distinctly: Upper 50 kDa (U50) in dark blue, Lower 50 kDa (L50) in wheat, the Converter in green, the IQ domain in cyan, switch-2 (Sw2) in orange, switch-1 (Sw1) in smudge green, Wedge in pale green.

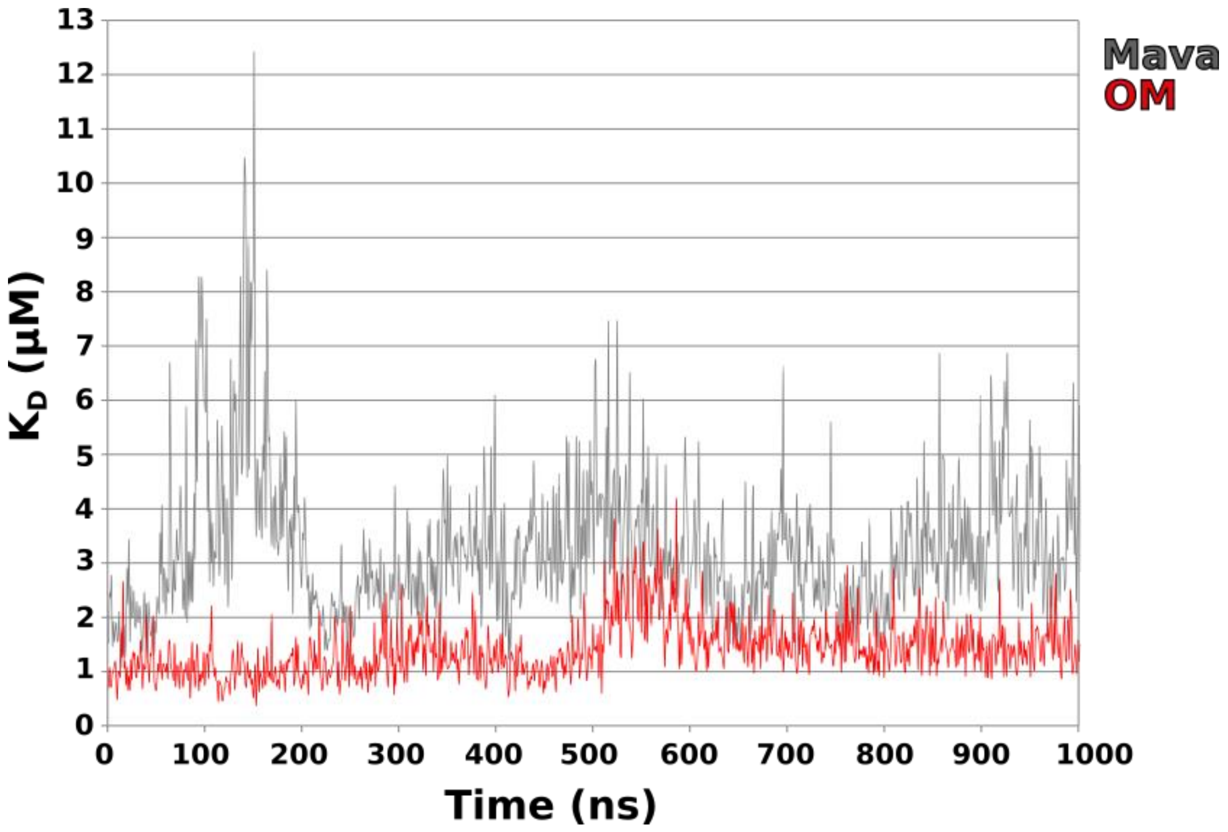

<u>Supplementary Figure 11 – Evolution of the dissociation constant (Kd) of OM and Mava during the time course of the simulations.</u> The binding constant was derived from the free energy calculations computed with PRODIGY-LIG[3,4]. During the OM MD simulation, the calculated Kd oscillates around ~1 µM. During the Mava MD simulations, the calculated Kd of the more mobile Mava goes through larger variations. Despite this variability, the average calculated Kd of Mava is 3.3 µM.

Supplementary Table 1 – Data collection and refinement statistics (molecular replacement)

	PPS-MD-Apo	PPS-S1-OM
**Data collection**		
Space group	$P4_122$	$P2_12_12_1$
Cell dimensions		
a, *b*, *c* (Å)	94.397, 94.397, 219.293	97.996, 122.503, 187.463
α, β, γ (°)	90.000, 90.000, 90.000	90.000, 90.000, 90.000
Resolution (Å)	94.397-2.759 (3.033-2.759)	102.547-1.928 (2.128-1.928)
R_{merge} (all I+ & I-)	0.273 (2.413)	0.153 (1.267)
R_{merge} (within I+ & I-)	0.296 (2.355)	0.139 (1.164)
Number of observations (total)	522201 (26600)	473345 (30533)
Number of observations (unique)	20166 (1009)	97819 (4891)
$I / \sigma I$	10.8 (1.5)	7.9 (1.5)
Completeness (Spherical) (%)	76.3 (15.8)	57.6 (11.3)
Completeness (Ellipsoidal) (%)	94.4 (64.2)	93.9 (73.7)
Redundancy	25.9 (26.4)	4.8 (6.2)
$CC_{1/2}$	0.999 (0.710)	0.991 (0.519)
**Refinement**		
Resolution (Å)	86.73-2.759 (2.858-2.759)	22.58-1.962 (2.032-1.962)
No. reflections	20165 (190)	92500 (636)
R_{work} / R_{free}	0.2010/0.2595	0.1874/0.2355
No. atoms	5723	15636
Protein	5687	14699
Ligand/ion	33	168
Water	3	769
B-factors	77.73	38.62
Protein	77.84	38.69
Ligand/ion	62.35	32.02
Water	45.18	38.60
R.m.s. deviations		
Bond lengths (Å)	0.016	0.048
Bond angles (°)	1.67	1.78

*Number of xtals for each structure should be noted in footnote. *Values in parentheses are for highest-resolution shell.

[AU: Equations defining various *R*-values are standard and hence are no longer defined in the footnotes.]
[AU: Ramachandran statistics should be in Methods section at the end of Refinement subsection.]
[AU: Wavelength of data collection, temperature and beamline should all be in Methods section.]

Supplementary Table 2 – Data collection, phasing and refinement statistics for PPS-S1-Mava and PPS-MD-Mava

	PPS-S1-Mava	PPS-MD-Mava
**Data collection**		
Space group	$P2_1$	$P2_12_12_1$
Cell dimensions		
a, *b*, *c* (Å)	102.801, 147.977, 116.977	68.577, 95.684, 127.139
α, β, γ (°)	90.000, 91.625, 90.000	90.000, 90.000, 90.000
Resolution (Å)	91.635-2.607 (2.859-2.607)	76.451-1.805 (1.986-1.805)
R_{merge} (all I+ & I-)	0.200 (1.212)	0.185 (2.562)
R_{merge} (within I+ & I-)	0.177 (1.111)	0.190 (2.498)
Number of observations (total)	313583 (18434)	1082068 (40777)
Number of observations (unique)	69889 (3494)	41812 (2083)
$I / \sigma I$	7.4 (1.5)	11.8 (1.3)
Completeness (Spherical) (%)	65.8 (13.7)	53.9 (10.9)
Completeness (Ellipsoidal) (%)	93.6 (61.2)	92.2 (64.6)
Redundancy	4.5 (5.3)	25.9 (19.6)
$CC_{1/2}$	0.985 (0.476)	0.999 (0.467)
**Refinement**		
Resolution (Å)	84.40-2.610 (2.703-2.610)	34.290-1.805 (1.869-1.805)
No. reflections	69880 (530)	41795 (75)
R_{work} / R_{free}	0.185/0.226	0.183/0.225
No. atoms	14993	6258
Protein	14383	5763
Ligand/ion	125	71
Water	485	711
B-factors	63.44	38.50
Protein	64.29	38.44
Ligand/ion	43.10	40.44
Water	43.28	39.01
R.m.s. deviations		
Bond lengths (Å)	0.014	0.015
Bond angles (°)	1.82	1.73

*Number of xtals for each structure should be noted in footnote. *Values in parentheses are for highest-resolution shell.

[AU: Equations defining various *R*-values are standard and hence are no longer defined in the footnotes.]
[AU: Ramachandran statistics should be in Methods section at the end of Refinement subsection.]
[AU: Wavelength of data collection, temperature and beamline should all be in Methods section.]

Supplementary Table 3 – Comparison of the interactions established by Mavacamten (Mava) and Omecamtiv mecarbil (OM).

Mava	β-cardiac myosin	OM
	$^{N\text{-term}}$Arg147	**(A)**
	$^{N\text{-term}}$Lys146	**(A)** electrostatic, carboxylate and εN-H
**(A), (B), (C)** stacking	$^{N\text{-term}}$Tyr164	**(A), (B), (C)** electrostatic, Carboxylate, water, hydroxy
**(B), (C)** electrostatic	$^{N\text{-term}}$Asp168	**(C)** electrostatic
	$^{N\text{-term}}$Glu170	**(D)** electrostatic, mediated by water.
**(A)**	^{Conv}Glu774	**(A)**
**(A), (B)**	^{Conv}Arg721	**(A), (B)**
**(A)**	^{Conv}Tyr722	**(B)**
**(A), (C)**	^{Conv}Leu770	**(B)**
	^{SH1}Pro710	**(D)**
**(C)** electrostatic	^{Conv}Asn711	**(C)** electrostatic, **(D)**
**(C)** electrostatic, backbone (CO)	^{Conv}Arg712	**(C)** electrostatic, backbone (CO) **(D)**
**(A), (B), (C)**	^{Conv}Ile713	**(B)**
**(C)**	$^{N\text{-term}}$Leu120	
**(C)**	^{Transducer}His666	**(D)** stacking
	^{Relay}His492	**(D)**

References

1. Liebschner, D. *et al.* Polder maps: improving OMIT maps by excluding bulk solvent. *Acta Crystallogr. Sect. D* **73**, 148–157 (2017).

2. Robert-Paganin, J., Auguin, D. & Houdusse, A. Hypertrophic cardiomyopathy disease results from disparate impairments of cardiac myosin function and auto-inhibition. *Nat. Commun.* **9**, 4019 (2018).

3. Vangone, A. *et al.* Large-scale prediction of binding affinity in protein-small ligand complexes: the PRODIGY-LIG web server. *Bioinformatics* **35**, 1585–1587 (2019).

4. Xue, L. C., Rodrigues, J. P., Kastritis, P. L., Bonvin, A. M. & Vangone, A. PRODIGY: a web server for predicting the binding affinity of protein-protein complexes. *Bioinformatics* **32**, 3676–3678 (2016).
