# Supplementary material for: Omecamtiv mecarbil and Mavacamten target the same myosin pocket despite opposite effects in heart contraction
Source: Nat Commun. 2024 Jun 7;15:4885. doi: 10.1038/s41467-024-47587-9 (PMC11161628; doi:10.1038/s41467-024-47587-9)
Supplement: Supplementary file 2 — Reporting Summary [file 41467_2024_47587_MOESM2_ESM.pdf]

nature portfolio

Corresponding author(s): Julien Robert-Paganin, Anne Houdusse

Last updated by author(s): 2024/02/22

# Reporting Summary

Nature Portfolio wishes to improve the reproducibility of the work that we publish. This form provides structure for consistency and transparency in reporting. For further information on Nature Portfolio policies, see our Editorial Policies and the Editorial Policy Checklist.

## Statistics

For all statistical analyses, confirm that the following items are present in the figure legend, table legend, main text, or Methods section.

| n/a | Confirmed | |
| --- | --- | --- |
| x | ☐ | The exact sample size (*n*) for each experimental group/condition, given as a discrete number and unit of measurement |
| x | ☐ | A statement on whether measurements were taken from distinct samples or whether the same sample was measured repeatedly |
| x | ☐ | The statistical test(s) used AND whether they are one- or two-sided<br>*Only common tests should be described solely by name; describe more complex techniques in the Methods section.* |
| x | ☐ | A description of all covariates tested |
| x | ☐ | A description of any assumptions or corrections, such as tests of normality and adjustment for multiple comparisons |
| x | ☐ | A full description of the statistical parameters including central tendency (e.g. means) or other basic estimates (e.g. regression coefficient) AND variation (e.g. standard deviation) or associated estimates of uncertainty (e.g. confidence intervals) |
| x | ☐ | For null hypothesis testing, the test statistic (e.g. *F*, *t*, *r*) with confidence intervals, effect sizes, degrees of freedom and *P* value noted<br>*Give P values as exact values whenever suitable.* |
| x | ☐ | For Bayesian analysis, information on the choice of priors and Markov chain Monte Carlo settings |
| x | ☐ | For hierarchical and complex designs, identification of the appropriate level for tests and full reporting of outcomes |
| x | ☐ | Estimates of effect sizes (e.g. Cohen's *d*, Pearson's *r*), indicating how they were calculated |

*Our web collection on statistics for biologists contains articles on many of the points above.*

## Software and code

Policy information about availability of computer code

| | |
| --- | --- |
| Data collection | No software was used for data collection. |
| Data analysis | -X-ray crystallography data processing with AutoProc (global, phasing, version 2020).<br><br>-Model refinement: Buster (global phasing, version 2020) and phenix suite (version 1.19.2).<br><br>-Molecular dynamics performed with GROMACS (version 2018.3).<br><br>-G_measures v.0.9d<br><br>-VMD version 1.9.4a53 (june 29, 2021)<br><br>-Pymol version 2.5.0 for the frame analysis.<br><br>-Coot version 0.9.2 |

For manuscripts utilizing custom algorithms or software that are central to the research but not yet described in published literature, software must be made available to editors and reviewers. We strongly encourage code deposition in a community repository (e.g. GitHub). See the Nature Portfolio guidelines for submitting code & software for further information.

## Data

Policy information about availability of data

All manuscripts must include a data availability statement. This statement should provide the following information, where applicable:

- Accession codes, unique identifiers, or web links for publicly available datasets
- A description of any restrictions on data availability
- For clinical datasets or third party data, please ensure that the statement adheres to our policy

The atomic models and the structure factors of PPS-MD-Apo, PPS-S1-Mava, PPS-MD-Mava and PPS-S1-OM are available at the PDB under the accession numbers 8QYP [https://www.rcsb.org/structure/8QYP], 8QYQ [https://www.rcsb.org/structure/8QYQ], 8QYR [https://www.rcsb.org/structure/8QYR] and 8QYU [https://www.rcsb.org/structure/8QYQU] respectively. Source data for all the graphs analyzing are provided as Source Data file.

## Research involving human participants, their data, or biological material

Policy information about studies with human participants or human data. See also policy information about sex, gender (identity/presentation), and sexual orientation and race, ethnicity and racism.

| | |
|---|---|
| Reporting on sex and gender | n/a |
| Reporting on race, ethnicity, or other socially relevant groupings | n/a |
| Population characteristics | n/a |
| Recruitment | n/a |
| Ethics oversight | n/a |

Note that full information on the approval of the study protocol must also be provided in the manuscript.

# Field-specific reporting

Please select the one below that is the best fit for your research. If you are not sure, read the appropriate sections before making your selection.

☒ Life sciences ☐ Behavioural & social sciences ☐ Ecological, evolutionary & environmental sciences

For a reference copy of the document with all sections, see nature.com/documents/nr-reporting-summary-flat.pdf

# Life sciences study design

All studies must disclose on these points even when the disclosure is negative.

| | |
|---|---|
| Sample size | In molecular dynamics, we analyzed three conditions: cardiac myosin S1 fragment without drug (Apo), complexed with Mavacamten (Mava) and complexed with Omecamtiv mecarbil (OM). The total duration of the simulations were 720 ns for Apo; 1020 ns for OM and Mava. |
| Data exclusions | No data were excluded, all the data from each simulation were used and analyzed (see Fig. 4 and 5). |
| Replication | The simulations were performed at least twice for each condition in order to ensure the reproducibility of the phenomenon phenomena discussed in this work. OM and Mava were performed twice and Apo three times. |
| Randomization | Randomization cannot be applied to molecular dynamics or structural biology experiments. |
| Blinding | Blinding cannot be applied to molecular dynamics or structural biology experiments. |

# Reporting for specific materials, systems and methods

We require information from authors about some types of materials, experimental systems and methods used in many studies. Here, indicate whether each material, system or method listed is relevant to your study. If you are not sure if a list item applies to your research, read the appropriate section before selecting a response.

## Materials & experimental systems

| n/a | Involved in the study |
| --- | --- |
| ☒ | ☐ Antibodies |
| ☒ | ☐ Eukaryotic cell lines |
| ☒ | ☐ Palaeontology and archaeology |
| ☒ | ☐ Animals and other organisms |
| ☒ | ☐ Clinical data |
| ☒ | ☐ Dual use research of concern |
| ☒ | ☐ Plants |

## Methods

| n/a | Involved in the study |
| --- | --- |
| ☒ | ☐ ChIP-seq |
| ☒ | ☐ Flow cytometry |
| ☒ | ☐ MRI-based neuroimaging |

# Plants

| | |
| --- | --- |
| Seed stocks | n/a |
| Novel plant genotypes | n/a |
| Authentication | n/a |
