# Supplementary material for: Omecamtiv mecarbil and Mavacamten target the same myosin pocket despite opposite effects in heart contraction
Source: Nat Commun. 2024 Jun 7;15:4885. doi: 10.1038/s41467-024-47587-9 (PMC11161628; doi:10.1038/s41467-024-47587-9)
Supplement: Supplementary file 4 — Description of Additional Supplementary Files [file 41467_2024_47587_MOESM4_ESM.pdf]

Supplementary Movie 1 – Comparison of the conformation of the Lever arm in the different structures.
PPS-S1-Mava is colored in cyan. PPS-S1-OM is colored in yellow. PPS-MD-Apo is colored in orange. For comparison, the last helix of the Converter from the PR state (PDB code 6FSA[2]) is shown. The specific position of Ala767 is colored in red and represented as stick.

Supplementary Movie 2 – Molecular dynamics of the PPS-S1-Apo condition (conformations explored without drug bound).
Different views are presented: the overall view to appreciate the dynamics of the Lever arm; the drug binding pocket (drug targeted site) in two orientations; the catalytic site. The subdomains are colored differently: N-terminal extension is colored in pink; N-terminal subdomain in grey; U50 in dark blue; L50 in light orange; Relay in yellow; SH1-helix in red; Converter in green; IQ region in cyan; ELC in light pink. Switch-1 (bright pink), Switch-2 (orange) are two connectors close to the catalytic site.

Supplementary Movie 3 – Molecular dynamics of the PPS-S1-OM (conformations explored when OM is bound).
Different views are presented: the overall view to appreciate the dynamics of the Lever arm; the drug binding pocket (drug targeted site) in two orientations; the catalytic site. The subdomains are colored differently: N-terminal extension is colored in pink; N-terminal subdomain in grey; U50 in dark blue; L50 in light orange; Relay in yellow; SH1-helix in red; Converter in green; IQ region in cyan; ELC in light pink. Switch-1 (bright pink), Switch-2 (orange) are two connectors close to the catalytic site.

Supplementary Movie 4 – Molecular dynamics of the PPS-S1-Mava (conformations explored when Mava is bound).
Different views are presented: the overall view to appreciate the dynamics of the Lever arm; the drug binding pocket (drug targeted site) in two orientations; the catalytic site. The subdomains are colored differently: N-terminal extension is colored in pink; N-terminal subdomain in grey; U50 in dark blue; L50 in light orange; Relay in yellow; SH1-helix in red; Converter in green; IQ region in cyan; ELC in light pink. Switch-1 (bright pink), Switch-2 (orange) are two connectors close to the catalytic site.

Supplementary Movie 5 – Dynamics of OM in the drug binding pocket during the time course of the molecular dynamics simulation.
The movie compares the initial position (structure, orange) of PPS-OM-S1 to the evolution during the time course of the dynamics (green). The nucleotide (yellow) and OM (dark blue) are represented as spheres.

Supplementary Movie 6 – Dynamics of Mava in the drug binding pocket during the time course of the molecular dynamics simulation.
The movie compares the initial position (structure, orange) of PPS-Mava-S1 to the evolution during the time course of the dynamics (green). The nucleotide (yellow) and Mava are represented as spheres. Mava is colored in dark blue in the initial position (structure) and in yellow in the dynamics.

Supplementary Movie 7 – Dynamics of OM and Mava in the drug binding site.
The movie compares how OM and Mava explore the drug binding pocket during the time

course of the dynamics. The two movies were synchronized to compare the positions at the same time.
